# Research on the Adhesive Strength of Different Bonding Structures for Modular Wind Turbine Blades

**DOI:** 10.3390/ma19040735

**Published:** 2026-02-14

**Authors:** Junpeng Yang, Afang Jin, Junhan Li, Fengrong Li

**Affiliations:** College of Mechanical Engineering, Xinjiang University, Ürümqi 830047, China

**Keywords:** CFRP laminate, adhesive joints, modular blades, damage evolution

## Abstract

To address the manufacturing and transportation challenges of large wind turbine blades, adhesive joints in modular blades have become a research focus. This study investigates six typical adhesive joint configurations for CFRP laminates using quasi-static three-point bending experiments and cohesive-zone finite element simulations. The adhesive interface is modeled with a bilinear traction–separation law using zero-thickness cohesive elements to capture damage initiation and propagation. Among the six designs, the double-slope joint exhibits the best static performance, achieving the highest peak load of 1017.26 N and showing delayed damage evolution. The superiority of the double-slope design is further examined at the blade level via a numerical cantilever model of equal-section modular blade segments under flapwise and edgewise loading. The predicted peak loads reach 9.82 × 10^7^ N (flapwise) and 5.51 × 10^7^ N (edgewise), ranking the double-slope joint highest among the investigated configurations. The findings show that the double-slope joint improves load capacity and resistance to degradation, and reveal failure mechanisms of adhesive joints, providing theoretical support for blade structural design.

## 1. Introduction

Driven by the large-scale development of the wind power industry, the large-scale design of megawatt wind turbine blades has become a core direction of technological iteration [1]. To overcome transportation limits and reduce lifecycle cost, segmented blade connections based on modular manufacturing has attracted increasing research attention [2,3,4]. At present, modular blade connections are mainly divided into mechanical joints and adhesive joints. In engineering practice, T-bolt joints [5] and embedded insert stud systems have become mainstream due to their high load efficiency and assembly reliability. However, as blade length approaches 100 m, the inherent drawbacks of mechanical joints become more evident, including high mass density, severe local stress concentration, and difficulty in corrosion protection. The negative effect of additional mass on overall performance has seriously limited their application in ultra-large blades. In contrast, adhesive bonding technology is developing rapidly [6] and has already been widely applied in more than one hundred aircraft. Critical components such as the fuselage, wings, and engines partly rely on adhesive bonding processes [7]. This indicates that adhesive joints have clear advantages in large composite structures and provide valuable reference for the structural design of wind turbine blades.

Compared with mechanical joints, adhesive joints show multi-dimensional technical advantages [8,9,10]. First, by eliminating metallic fasteners, structural lightweighting can be achieved, which reduces blade mass and improves aerodynamic efficiency and power generation performance. Second, the continuous stress transfer at the adhesive interface alleviates local stress concentration and decreases the probability of fatigue crack initiation. Its damage tolerance also helps delay crack growth. Third, the viscoelasticity of the adhesive layer provides excellent energy dissipation, while its non-metallic nature enhances resistance to salt spray and humid heat. These characteristics are of great importance for the long-term service of offshore wind turbine blades under harsh environments [11]. In summary, adhesive bonding, with the advantages of its light weight, fatigue resistance, and corrosion resistance, is regarded as a key connection scheme for large blades and offshore wind development.

Despite these advantages, systematic studies on adhesive joints in wind turbine blades are still limited, whereas adhesive joints of carbon fiber-reinforced polymer (CFRP) laminates have accumulated rich theoretical results [12]. Owing to its high specific strength, low density, and excellent corrosion resistance, CFRP has been widely used as a key load-bearing material in the main spar regions of large wind turbine blades for lightweight structural design [13]. Therefore, this study takes CFRP laminates as the research entry point. In current studies, Adin [14] used ANSYS to analyze the effect of adherend size and adhesive angle on the performance of bonded joints under tensile load. Akpinar et al. [15] tested single-lap joints of woven composite laminates bonded with adhesives containing different nanoparticles, and they found that nanoparticles improved the mechanical performance. Sun et al. [16] investigated the effect of scarf angles on tensile behavior and failure modes using experiments and numerical models, and compared shear stress distributions between small and large scarf angles. Khan et al. [17] established design rules based on experimental data and finite element modeling, and analyzed the influence of adhesive type and thickness on double-lap joint strength, as well as the related failure surfaces and modes. Davaasambuu [18] analyzed various traditional adhesive joints and reviewed their differences in load transfer, stress distribution, fatigue resistance, and energy absorption under impact loading. He also highlighted that a geometry–material mismatch strongly affects stress concentration and failure modes. In the broader context, CFRP joining has been widely investigated for aerospace and other composite structures over the past decades, and classic bonded-joint configurations (e.g., single-/double-lap, scarf, and stepped joints) have been studied extensively. It is well recognized that joint geometry, bondline thickness, and the peel/shear stress concentrations near overlap ends largely govern joint strength and failure modes. Moreover, progressive damage in bonded composites often involves not only interfacial debonding and cohesive failure of the adhesive, but also concurrent ply-level failures (e.g., matrix cracking and delamination), and cohesive-zone-based numerical approaches have been widely adopted to capture crack initiation and propagation under different loading conditions. However, transferring these insights directly to modular wind-turbine blades remains non-trivial, because blade segmentation requires joints to be integrated without altering the external aerodynamic profile and to operate under bending-dominated service conditions where mixed-mode interfacial stresses can be critical. In addition, the structural response must be evaluated at the blade segment scale rather than only at the coupon level.

**Figure 1:**
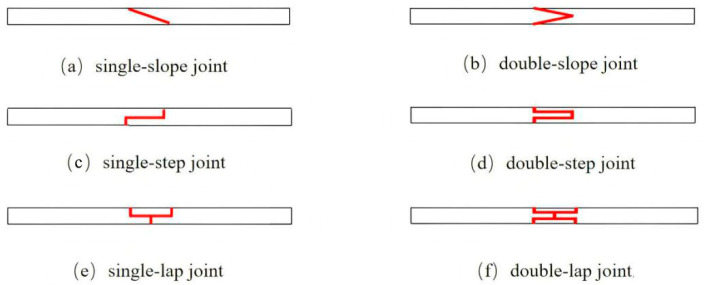
Six typical adhesive joint configurations.

These findings indicate that each joint type has specific applications. Thus, without altering the external aerodynamic profile of blades, several typical adhesive joints can be adopted in modular blades, as shown in Figure 1: (a) single-slope joint, (b) double-slope joint, (c) single-step joint, (d) double-step joint, (e) single-lap joint, and (f) double-lap joint.

The development of large wind turbine blades not only improves wind energy efficiency and reduces power generation cost but also plays a key role in energy transition and technological innovation. It is of great importance for mitigating climate change and achieving sustainable development [19,20]. However, the literature review shows that systematic studies on adhesive joints of modular blades are still limited. Most results focus on patents of connection configuration and assembly [21,22,23,24], while systematic theoretical and experimental studies in journals and conferences are lacking. Therefore, in-depth studies on adhesive joints of modular blades are urgently needed. This paper proposes a failure simulation method for adhesive interfaces of modular blade subcomponents. Cohesive layer damage is simulated numerically and validated by experiments, and the corresponding displacement–load data are obtained. On this basis, multiscale finite element modeling and experimental comparison are carried out for CFRP laminate adhesive joints. Accordingly, this work establishes a modeling-and-validation route that links coupon-level CFRP laminate tests with blade segment simulations for six representative joint geometries, enabling a comparative assessment of load capacity, damage evolution, and failure mechanisms under flapwise and edgewise bending. A numerical method is established for adhesive joints of composite wind turbine blades. Furthermore, failure simulation of adhesive interfaces is conducted using a cohesive zone model based on fracture mechanics. Interface failure modes and ultimate loads are obtained, and a finite element method for adhesive joints of blades is derived. This provides theoretical support for optimization and the reliability assessment of adhesive processes in megawatt wind turbine blades.

## 2. Numerical Simulation

### 2.1. CZM Formulation and Implementation for the Adhesive Layer

To describe damage initiation and propagation in the adhesive layer, a cohesive zone model (CZM) was employed in this study [25]. The adhesive behavior was represented by a bilinear traction–separation law, in which the interface exhibits linear elastic behavior prior to damage initiation and progressive stiffness degradation after damage onset. Damage initiation was governed by the maximum nominal stress (MAXS) criterion, assuming that damage initiates when either the normal or shear traction reaches its corresponding peak value.

Following damage initiation, an energy-based damage evolution law was adopted, characterized by the critical fracture energies in Mode I and Mode II. Mixed-mode fracture behavior was accounted for using an energy-based interaction criterion. The CZM parameters implemented for the Araldite 2015 [26] adhesive, including peak tractions and fracture energies, were adopted from the literature and are summarized in Table 1. It should be noted that these literature parameters were not re-calibrated through dedicated fracture tests for the specific bondline thickness, curing condition, and quasi-static loading rate adopted in this work. Given the known sensitivity of CZM predictions to peak traction and fracture energy, the present simulations are primarily used to compare the relative performance and damage evolution trends among the investigated joint geometries, rather than to claim a fully material-calibrated parameter set for all processing/geometry variations.

The cohesive behavior was implemented using zero-thickness cohesive elements embedded at the adhesive interface. To ensure an adequate resolution of the fracture process zone, a refined mesh was employed in the cohesive region, with a characteristic element size of 0.2 mm. This mesh resolution was selected to provide stable numerical predictions of peak load and damage evolution trends. All CZM simulations were performed under quasi-static loading conditions at room temperature, consistent with the experimental three-point bending tests conducted in this study. Although no dedicated fracture tests were performed to recalibrate the CZM parameters, the suitability of the adopted parameter set was indirectly validated through good agreement between numerical predictions and laminate-level experimental results across the different joint configurations.

### 2.2. Finite Element Modeling

Based on the ABAQUS (2023) finite element analysis platform, numerical models of carbon fiber-reinforced polymer (CFRP) adhesive joints and large wind turbine blades are constructed. To accurately predict the failure load threshold and progressive damage behavior of adhesive interfaces, the model adopts zero-thickness cohesive elements (traction–separation law) inserted at the adhesive interface to characterize the bonding behavior and simulate damage initiation and propagation in the adhesive layer. Intralaminar damage (e.g., matrix cracking) and explicit interlaminar delamination of the CFRP adherends were not modeled; thus, the cohesive damage (SDEG) is intended to capture the dominant interface fracture governing the global response up to peak load.

**Figure 2:**
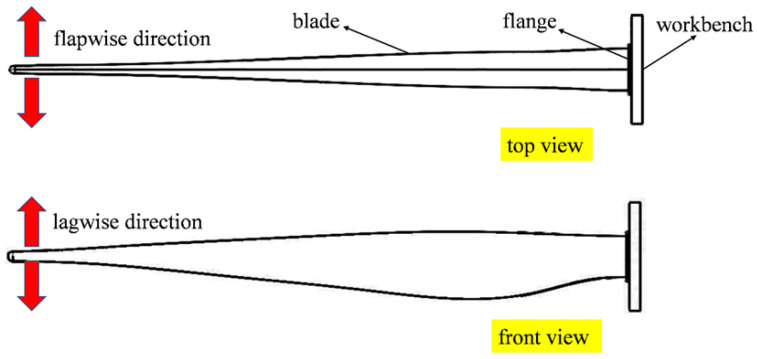
Flapwise and edgewise loading model of the blade.

According to the international standard IEC 61400-23 [27], full-scale blade static tests are mainly conducted in four directions: positive and negative flapwise and positive and negative edgewise. The flapwise direction refers to the plane of rotor rotation, while the edgewise direction is perpendicular to it (as illustrated in Figure 2). Static failure tests of blades are generally performed using the cantilever beam loading method. To better represent the actual operating conditions of blades, while also considering the feasibility of experiments and the generality of the results, the loading conditions are simplified in this study. Three-point bending loads are applied to CFRP laminate adhesive joints, while cantilever beam loading is applied to blades for flapwise and edgewise tests.

#### 2.2.1. Laminate Model

The specimen geometry is a laminate with dimensions of 150 mm × 25 mm × 4.8 mm, with the adhesive region located at the mid-plane and an effective bonding length of 20 mm. The laminate matrix is discretized using reduced integration solid elements (C3D8R). The adhesive interface is modeled using zero-thickness cohesive elements inserted at the bondline; therefore, the adhesive layer is not represented by a finite-thickness solid mesh. The cohesive elements are discretized with an in-plane mesh size of 1 mm × 1 mm (refined to 0.2 mm in the convergence study), consistent with the characteristic length of the fracture process zone. The final model consists of 39,156 nodes and 32,175 elements.

**Figure 3:**
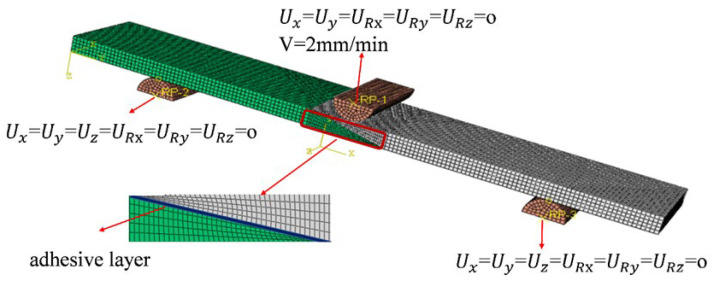
Three-point bending model of the laminate.

For the single-slope joint under three-point bending, the boundary conditions are defined as follows: all degrees of freedom are constrained (U_x_ = U_y_ = U_z_ = U_Rx_ = U_Ry_ = U_Rz_ = 0) at 20 mm from both end faces. A quasi-static displacement load along the gravity direction (*Z*-axis) is applied at the mid-span, 75 mm from the right end, at a loading rate of 2 mm/min. At the same time, the lateral displacement and rotational degrees of freedom are constrained (U_x_ = U_y_ = U_Rx_ = U_Ry_ = U_Rz_ = 0). The structural loading configuration is shown in Figure 3.

#### 2.2.2. Modular Blade Model

**Figure 4:**
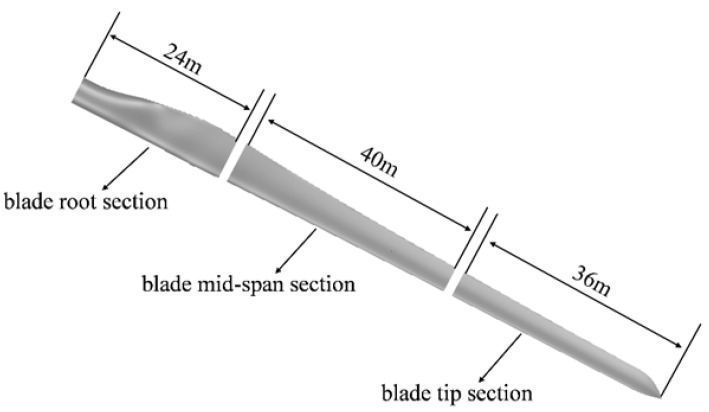
Segmentation positions of the blade.

In this study, a 10 MW blade with a length of 100 m, provided by a domestic blade manufacturer, is selected as the research object. Table 2 lists part of the technical parameters of the wind turbine blade. The segmentation position must first avoid high-stress regions and vibration-mode nodes, and regions with smooth airfoil curvature are preferred to maintain aerodynamic efficiency. Second, the position should strictly comply with transportation limits to ensure the feasibility of manufacturing tolerances and connection processes. Finally, a multi-objective balance is achieved among structural safety, aerodynamic performance, and economic cost. Based on these principles, the segmentation positions are preliminarily set at 24 m and 64 m from the blade root, as shown in Figure 4.

Because the geometric dimensions and composite layups of blades vary continuously along the spanwise and chordwise directions, establishing a full three-dimensional solid element model for an entire blade is extremely difficult and requires considerable computational time. Therefore, in this study, three-dimensional solid element models with equal cross-sections are established for blade segments before and after the segmentation points. Table 3 presents selected geometric and airfoil parameters of the blade.

According to the two preliminary segmentation points selected in this study, located 24 m and 64 m from the blade root, the blade at the 24 m segmentation point bears greater load stresses under different operating conditions. Therefore, the airfoil structure at the 24 m position is selected to establish an equal-section three-dimensional solid element model for analysis. Around the 24 m segmentation point, there are two primary control airfoils located at 22.3% and 24.8% of the blade span. In this study, the aerodynamic profile and layup structure at 24.8% are chosen for finite element simulation. The adopted airfoil is DU99-W-350. The blade uses a two-shear-web structural layout, and the chord length at this section is 4 m.

The material property data are taken from the DOE/MSU [28] database, as listed in Table 4. E_L_ and E_T_ represent the elastic modulus in the fiber direction and in-plane transverse to the fiber direction, respectively; G_LT_ is the in-plane shear modulus; _VLT_ is the in-plane Poisson’s ratio; T_FSL_ is the longitudinal tensile strength; C_FSL_ is the longitudinal compressive strength; and τ_TU_ is the shear strength. Assuming the composite is transversely isotropic, the elastic modulus in the thickness direction is taken as equivalent to the in-plane transverse modulus. The structural adhesive used is Araldite 2015, which has high shear and peel strength and is widely applied in the bonding of wind turbine blades. Note that two material systems are intentionally involved for different purposes. The coupon-level three-point bending tests were conducted on CFRP laminates (Table 5) to validate the interfacial damage modeling strategy and its ability to reproduce the load–displacement response and damage initiation trend. For the blade segment simulation, material properties were taken from the DOE/MSU reference blade database, representing a GFRP/foam blade section (Table 4) that is widely used as a benchmark in wind turbine blade research. Therefore, the blade segment analysis is trend-/ranking-oriented, focusing on the comparative performance and failure mechanisms among joint geometries, rather than a direct quantitative calibration or absolute-strength comparison with the CFRP coupon tests.

The mesh model of the segmented blade was created using Hypermesh (2021), where hexahedral solid elements were adopted for the composite skin and shear webs. After importing the mesh into ABAQUS, a bilinear cohesive model combined with zero-thickness hexahedral cohesive elements was used to predict adhesive layer failure. The equal-section blade model was discretized into 158,340 nodes and 110,859 elements.

**Figure 5:**
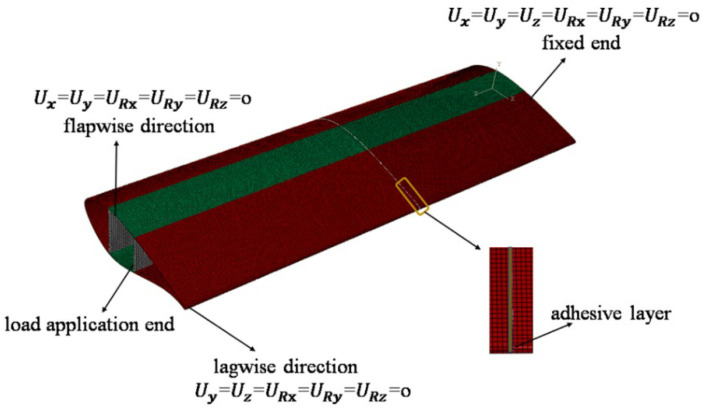
Cantilever beam model of the modular blade.

For the modular blade cantilever bending model in Figure 5, the boundary conditions are defined as follows. At the blade root on the right end, all degrees of freedom are constrained (U_x_ = U_y_ = U_z_ = U_Rx_ = U_Ry_ = U_Rz_ = 0). When a quasi-static displacement load is applied along the gravity direction (*Y*-axis) at the left end, the blade is in the flapwise configuration, with its lateral displacement and rotational degrees of freedom constrained (U_x_ = U_y_ = U_Rx_ = U_Ry_ = U_Rz_ = 0). When a quasi-static displacement load is applied along the direction perpendicular to gravity (*X*-axis) at the left end, the blade is in the edgewise configuration, with its lateral displacement and rotational degrees of freedom constrained (U_y_ = U_z_ = U_Rx_ = U_Ry_ = U_Rz_ = 0).

It should be noted that the adhesive joint geometry adopted in the modular blade segment follows the same orientation as that illustrated in Figure 1. For the cantilever model (Figure 5), the blade root at the right end was fully constrained, and the quasi-static displacement load was applied at the left end to represent the blade-tip loading. Accordingly, for direction-dependent configurations (e.g., the single-/double-slope and single-/double-step joints), only this root-fixed/tip-loaded orientation was considered in the present study. The mirrored orientation obtained by swapping the fixed and loaded ends was not simulated; therefore, a direct comparison of peak load and failure initiation between the two orientations is not reported here. Considering the asymmetric joint geometry and cantilever boundary condition, the mirrored orientation may modify the local peel/shear stress distribution and thus affect the peak load, which will be investigated in future work.

### 2.3. Mesh Size Convergence Analysis

**Figure 6:**
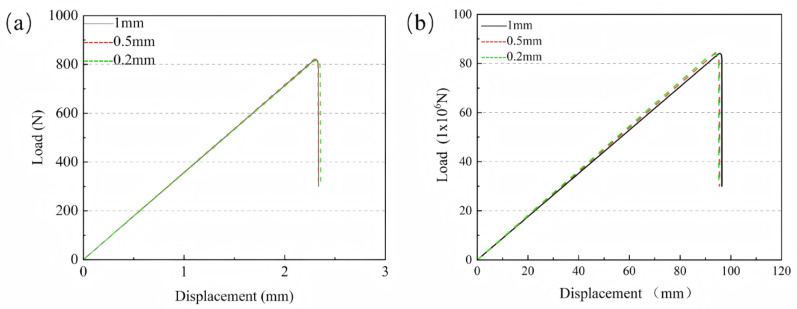
Load–displacement curves of (**a**) the modular blade and (**b**) the CFRP laminate.

To analyze the convergence of mesh size, the peak load variations in the CFRP laminate model with a single-slope adhesive joint and the modular blade model under flapwise loading were compared. In this study, the mesh size of the adhesive layer region was set to 1 mm, then refined to 0.5 mm, and further refined to 0.2 mm. The peak loads of the CFRP laminate model with different mesh sizes were 818.535 N, 822.682 N, and 816.467 N, respectively. For the modular blade model, the peak loads with different mesh sizes were 83.46 × 10^6^ N, 84.64 × 10^6^ N, and 83.83 × 10^6^ N, respectively. Figure 6a,b show the displacement curves of the modular blade and CFRP laminate, respectively. It can be observed that the peak load and failure displacement generally converge to stable values, and the relative differences in peak load are small.

## 3. Analysis of CFRP and Modular Blade Connection Performance

### 3.1. Adhesive Strength Analysis of CFRP Laminates

#### 3.1.1. Simulated Failure Load

**Figure 7:**
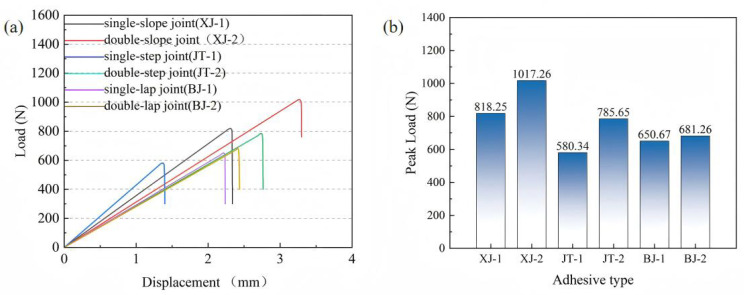
Mechanical performance of different adhesive structures: (**a**) load–displacement curves; (**b**) bar chart of peak loads.

This section aims to clarify the mechanical behavior of six typical adhesive structures of CFRP laminates under bending loads through numerical simulations based on the established finite element models. For clarity, the abbreviations used in Figure 7 are defined as follows: XJ denotes slope joints (XJ-1: single-slope joint; XJ-2: double-slope joint), JT denotes step joints (JT-1: single-step joint; JT-2: double-step joint), and BJ denotes lap joints (BJ-1: single-lap joint; BJ-2: double-lap joint). Figure 7a shows the load–displacement curves of CFRP laminate specimens with different adhesive structures under three-point bending. In general, as displacement increases, the load–displacement curve of the CFRP laminates exhibits a linear relationship in the initial stage. When the peak load is reached, the CFRP laminates can no longer withstand the external load and fail suddenly.

**Figure 8:**
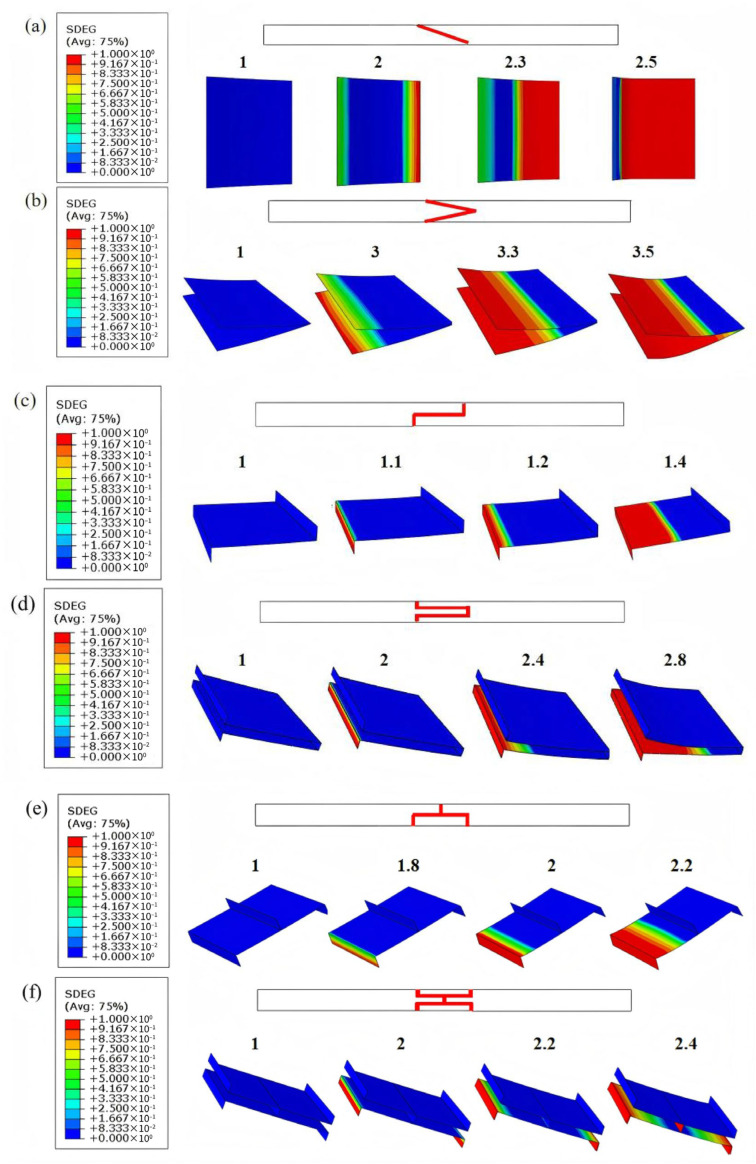
Damage evolution of adhesive layers in different structures: (**a**) single-slope joint, (**b**) double-slope joint, (**c**) single-step joint, (**d**) double-step joint, (**e**) single-lap joint, and (**f**) double-lap joint.

In the three-point bending static loading tests of CFRP laminates, the peak loads of different adhesive structures show clear differences. As shown in Figure 8b, the double-slope joint exhibits the highest load-bearing capacity, reaching a peak of 1017.26 N, which is about 24.3% higher than that of the single-slope joint. This indicates that the double-slope geometry can effectively optimize the stress transfer path and significantly improve the overall load-bearing performance. The double-step joint shows a peak load of 785.65 N, which is also notably higher than that of the single-step joint, suggesting that step-type structures are likewise beneficial for improving stress distribution. In contrast, the improvement of lap joints is relatively small, which may be attributed to their obvious stress concentration and similar interfacial peeling modes under three-point bending. These results demonstrate that geometric optimization of adhesive interfaces directly affects the bending capacity of CFRP laminates. Among them, slope joints are particularly effective, as they promote more uniform stress distribution and reduce stress concentration, thereby enhancing structural strength. Therefore, the observed superiority of the double-slope joint should be interpreted as valid for the present geometric set; different slope angles or overlap lengths may further change the stress distribution and peak load.

#### 3.1.2. Damage Evolution

Considering that instantaneous failure during experiments is insufficient to capture the initiation and evolution of damage in CFRP laminates, this section uses the established FE model to numerically simulate the failure process of adhesive layers. Within the ABAQUS finite element framework, the scalar stiffness degradation variable (SDEG) [29,30] is employed as the core internal variable in the continuum damage mechanics model. It quantitatively characterizes the degree of macroscopic stiffness degradation induced by the evolution of microscopic defects. The SDEG is a scalar ranging from 0 to 1: a value of 0 represents an intact material point, a value of 1 corresponds to complete failure, and intermediate values reflect partial damage states and the associated proportion of stiffness reduction.

Figure 8 illustrates the damage evolution contours of the adhesive layer for six different structures under varying load–displacement conditions. For each structure, the load–displacement level corresponding to each contour is explicitly indicated in the figure, so as to identify the deformation stage at which the damage state is extracted and to facilitate comparison and reproducibility. All structures show a gradual increase in damage with external loading. The overall damage process is nonlinear, with both the rate and extent of damage propagation increasing as the load rises. The spatial distribution of damage varies among structures. Some structures exhibit localized regions of concentrated damage, indicated by pronounced red zones, while others show more uniform distributions.

From the evolution of the SDEG, it can be observed that slope joints exhibit relatively uniform crack propagation along the interface during damage evolution. They maintain higher load-bearing capacity at larger deflections and display more stable failure modes. Step joints, through geometric transitions, effectively mitigate end-stress concentrations, slowing the damage propagation rate and providing moderate bearing capacity. In contrast, lap joints experience significant peel stresses at the ends, leading to rapid crack initiation and fast penetration through the adhesive layer. As a result, premature global failure occurs, and both load-bearing and deformation capacities are the weakest.

From an engineering standpoint, the SDEG contours further indicate different levels of joint durability, inspection focus, and tolerance sensitivity. The slope joints show more distributed and stable damage development, implying improved robustness to stress concentration and to moderate manufacturing scatter (e.g., bondline thickness non-uniformity). Step joints exhibit damage interactions near geometric transitions, suggesting higher sensitivity to local alignment/edge finishing and thus requiring stricter fabrication control. For lap joints, the strongly end-localized damage identifies the overlap ends as the critical zones; therefore, quality control and in-service inspection should prioritize these regions.

These findings are consistent with the previously obtained load–displacement curves and peak load results under three-point bending, indicating that geometric design plays a decisive role in controlling adhesive layer damage evolution, delaying failure, and enhancing overall load-bearing capacity.

#### 3.1.3. Specimen Fabrication

In this work, the CFRP laminate specimens were fabricated using T300 carbon fiber composites produced by a carbon fiber technology company in Jiangsu, China. This composite adopts a non-woven design, and the prepregs were cured and molded in an autoclave under high temperature and high pressure. The material has a density of 1.76 g/cm^3^ with 32 plies in a symmetric layup sequence of [45/0/90/−45]_4_s, and the thickness of each ply is 0.15 mm. The mechanical property parameters of the T300 CFRP laminate are listed in Table 5 [31].

The adherends with different joint geometries were manufactured by CNC machining to ensure dimensional accuracy and to avoid thermal damage on the bonding surfaces. After machining, the bonding areas were abraded with 400-grit sandpaper as a surface treatment to increase surface roughness and enhance interfacial bonding strength. The CFRP laminate surfaces, especially the bonding region, were then cleaned with acetone to remove contaminants prior to adhesive application.

**Figure 9:**
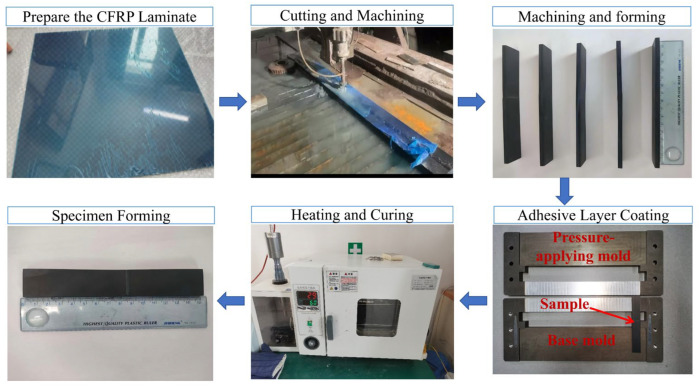
Preparation process of CFRP laminate samples.

An epoxy-based structural adhesive, Araldite 2015, was used for bonding, and it was prepared as a two-component system. The adhesive was mixed at a 1:1 volume ratio and thoroughly stirred until a homogeneous mixture was obtained. During bonding, a customized thickness control fixture, together with a dedicated forming procedure, was employed to precisely control the adhesive layer thickness. Specifically, after applying the adhesive, the assembly was placed into the fixture: the adherend was first positioned in the lower half of the mold, ensuring that its upper surface was flush with the mold edge. Two pre-set thickness probes were then placed on both sides of the bonded region, followed by closing and fixing the upper mold half. The assembled fixture was subsequently transferred to a temperature-controlled drying oven for curing. During thermal curing, the adhesive expanded and came into contact with the probes, thereby defining and maintaining the final bondline thickness; the probe thickness directly determined the cured adhesive layer thickness. Accordingly, the nominal adhesive layer thickness was maintained at 0.2 mm to ensure reproducibility across different joint geometries. After assembly, the bonded specimens were cured at 60 °C for 2 h, followed by cooling at room temperature for 2 h before subsequent testing. Figure 9 depicts the fabrication process of the CFRP laminate specimens.

#### 3.1.4. Experimental Validation

**Figure 10:**
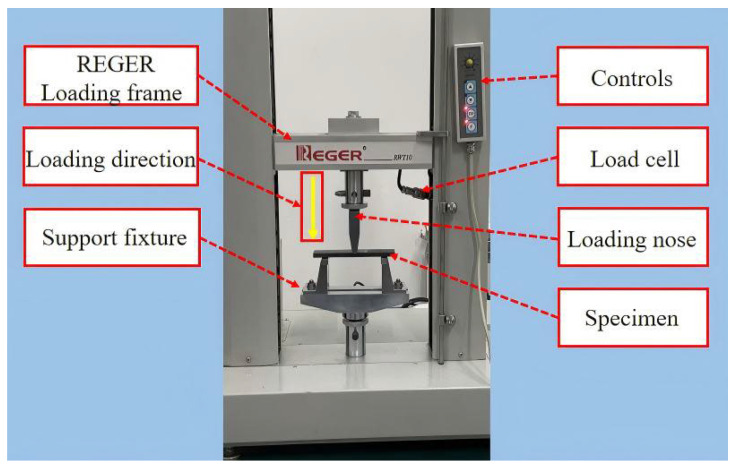
Three-point bending test setup, including the loading nose and bottom support fixture.

Based on the studies in Section 3.1.1 and Section 3.1.2 of six typical adhesive structures under bending loads, this section presents experimental investigations on the mechanical performance and failure modes of different adhesive structures under bending conditions. In this study, three-point bending tests of CFRP laminate specimens were conducted under a displacement-controlled mode. Following the ASTM D7264/D7264M-21 standard [32], all specimens were tested using a 50 kN electronic universal testing machine, as shown in Figure 10. During the tests, the specimen was supported by a bottom support fixture, and the load was applied by the testing machine actuator through the loading nose. The tests were performed at a constant crosshead displacement rate of 2 mm/min. Load, displacement, and time were recorded simultaneously using the built-in data acquisition system with a sampling frequency of 20 Hz to ensure data continuity. All tests were carried out at room temperature. For each loading case, at least five specimens were tested to reduce experimental scatter, and two specimens showing the largest deviations from the average ultimate failure load were excluded during data analysis, resulting in three valid results for each adhesive joint configuration.

**Figure 11:**
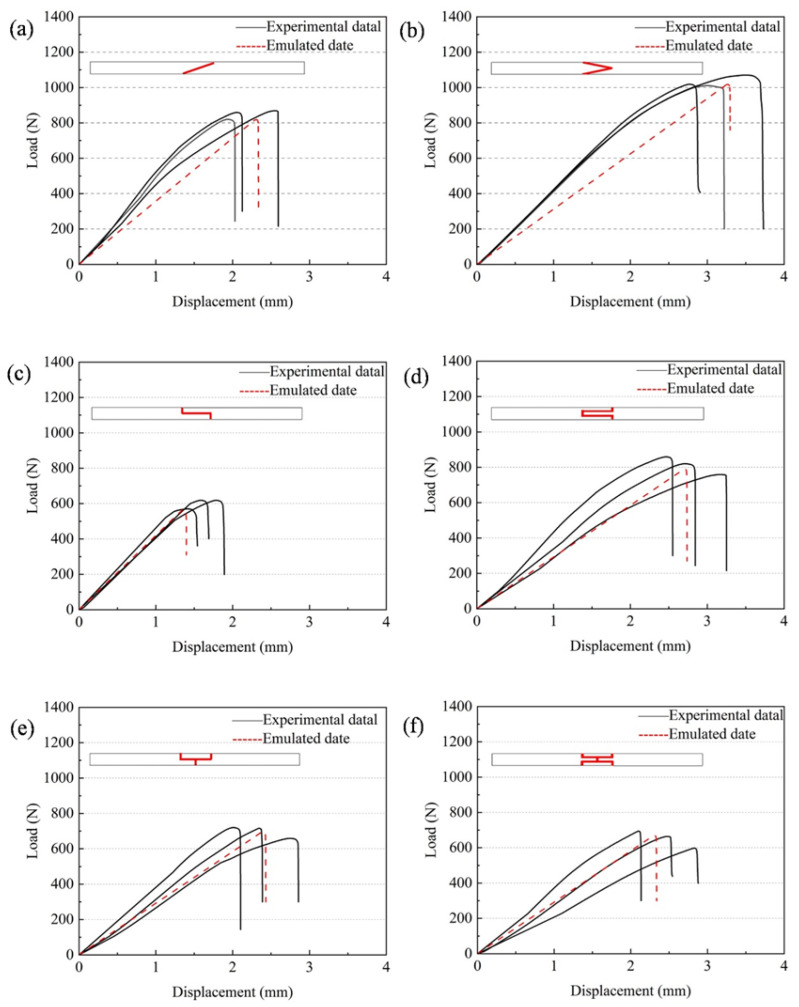
Load–displacement curves of CFRP laminates with different adhesive structures: (**a**) single-slope joint, (**b**) double-slope joint, (**c**) single-step joint, (**d**) double-step joint, (**e**) single-lap joint, and (**f**) double-lap joint.

All load–displacement curves of CFRP laminates considered in this study were obtained from quasi-static bending tests. Figure 11a–f shows the load–displacement curves of CFRP laminates with different adhesive structures. The results indicate that the errors of all models are within 10%, which means that the FEM provides reliable and accurate predictions of the mechanical behavior of CFRP laminates with different joint configurations under bending loads.

Overall, good agreement is observed in the elastic stage, accurately reflecting the initial stiffness. At the peak load and failure stage, the simulation results are generally slightly lower than the experimental values, and the descending branches appear more idealized. In contrast, the experimental curves exhibit some scatter due to specimen variability. It should be noted that the experiments show more complicated fracture features, whereas the numerical model mainly captures interfacial debonding through the cohesive-zone formulation. Therefore, the present agreement should be interpreted primarily in terms of the global load–displacement response and the peak load, which are dominated by the onset and propagation of interfacial damage, even though additional failure modes (e.g., matrix cracking and delamination) may coexist in the tests. The more idealized post-peak response in the simulations is thus expected, because these secondary damage mechanisms and their interactions are not explicitly represented. In this sense, the comparison remains valid for evaluating the relative load-bearing performance among joint configurations, rather than reproducing every fracture detail observed experimentally.

The comparison among different adhesive structures reveals the same trend as in the simulations. Slope-type joints perform better than step-type and lap-type joints, especially the double-slope joint, which reaches a peak load of 1017.26 N, significantly higher than other adhesive structures, highlighting its superiority under the combined action of bending and peeling resistance.

#### 3.1.5. Failure Modes

**Figure 12:**
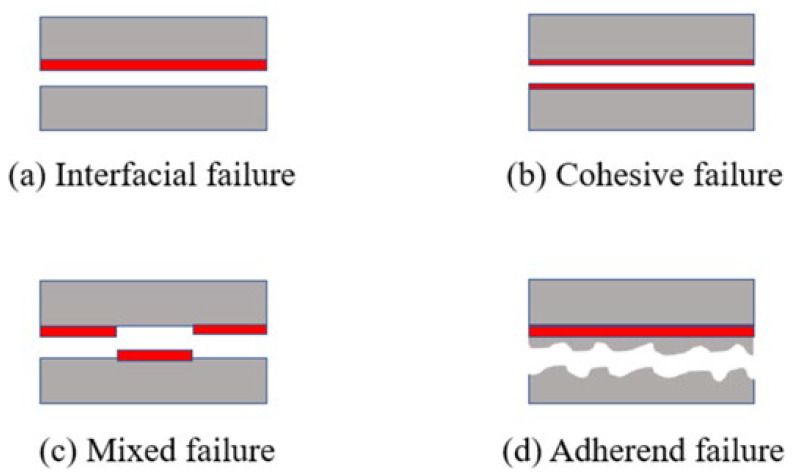
Main failure modes of adhesive joints.

The main failure modes of CFRP laminate adhesive joints can be classified into four types—interfacial failure, cohesive failure, mixed failure, and adherend failure—as seen in Figure 12. Interfacial failure occurs at the interface between the adhesive and the laminate and is considered the least desirable mode. Cohesive failure indicates that the adhesive performance is fully utilized, with the fracture occurring entirely within the adhesive layer and the adhesive remaining on both fracture surfaces; this is regarded as a more favorable mode. Mixed failure is the most common in practical applications, typically initiated by interfacial debonding at the adhesive ends or cohesive cracking within the adhesive layer. Adherent failure is also significant, manifested as matrix cracking, delamination, or other structural damage of the laminate.

**Figure 13:**
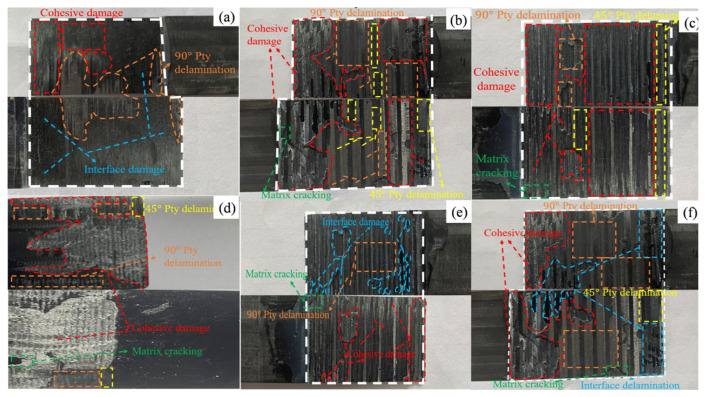
Fracture morphologies of CFRP joints with different adhesive designs: (**a**) single-lap joint; (**b**) double-lap joint; (**c**) single-slope joint; (**d**) double-slope joint; (**e**) single-step joint; (**f**) double-step joint.

As shown in Figure 13, under three-point bending loads, the fracture surfaces of CFRP joints with different adhesive structures exhibit typical mixed failure modes, but the dominant failure mechanisms differ. For the single-lap joint Figure 13a, the fracture is mainly characterized by interfacial debonding, with smooth fracture surfaces and little adhesive residue, indicating that the weak point is concentrated at the bonding interface. For the double-lap joint in Figure 13b, the fracture morphology is more complex: in addition to interfacial delamination, significant cohesive failure within the adhesive layer is observed, with adhesive residues remaining on the laminate matrix, suggesting that both the adhesive layer and the interface contribute to failure.

For the single-slope joint, Figure 13c, the fracture is dominated by cohesive failure of the adhesive layer, accompanied by resin cracking in the laminate matrix. This indicates that the bonding strength exceeds the matrix strength, leading to failure to propagate inside the laminate rather than being limited to the interface or adhesive layer. For the double-slope joint, Figure 13d, cohesive failure of the adhesive layer is dominant, while local delamination occurs in the 90° direction. Distinct cohesive damage within the adhesive layer demonstrates strong interfacial bonding, with failure concentrated primarily in the adhesive.

For the single-step joint, Figure 13e, the fracture morphology is more complex, with cohesive failure, interfacial debonding, and matrix resin cracking occurring together, showing a multi-path fracture mechanism. In contrast, for the double-step joint, Figure 13f, after progressive failure of the adhesive layer, significant delamination and matrix cracking occur, and part of the load is borne by the laminate itself. Overall, the failure modes of the adhesive structures show strong spatial coupling, where cohesive failure, interfacial debonding, delamination propagation, and matrix cracking jointly dominate the final failure process

### 3.2. Strength Analysis of Modular Blade Adhesive Joints

#### 3.2.1. Simulated Failure Load

**Figure 14:**
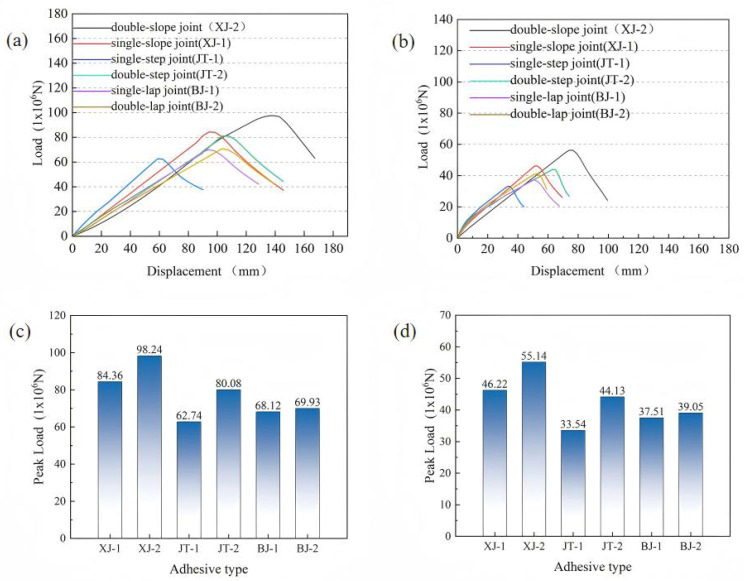
Performance of modular blades with different adhesive joints: (**a**) load–displacement curves under flapwise loading; (**b**) load–displacement curves under edgewise loading; (**c**) bar chart of peak loads under flapwise loading; (**d**) bar chart of peak loads under edgewise loading.

Based on the study in Section 3.1 of the mechanical performance and failure mechanisms of six typical CFRP laminate joints, this section focuses on revealing the mechanical behavior of modular blades with different adhesive joints under various loading conditions. For clarity, the abbreviations used in Figure 14 are defined as follows: XJ denotes slope joints (XJ-1: single-slope joint; XJ-2: double-slope joint), JT denotes step joints (JT-1: single-step joint; JT-2: double-step joint), and BJ denotes lap joints (BJ-1: single-lap joint; BJ-2: double-lap joint). Figure 14a shows the load–displacement curves of modular blades with different adhesive structures in the flapwise direction, while Figure 14b presents the curves in the edgewise direction. In general, as displacement increases, the load–displacement curve of the modular blade exhibits a linear relationship in the initial stage. When the applied load reaches its peak, the blade can no longer withstand the external load and gradually fails.

Figure 14c,d show that the peak loads of different adhesive structures vary significantly under the two loading conditions. In the flapwise case, the double-slope joint exhibits the highest load-bearing capacity, reaching 98.24 × 10^6^ N, which is much higher than that of the single-slope joint (84.36 × 10^6^ N). This indicates that the double-slope geometry provides a clear advantage in stress distribution. The double-step joint also shows better performance than the single-step joint, while the difference between the double-lap and single-lap joints is relatively small. These results suggest that geometric optimization can significantly improve the load-bearing capacity of slope-type and step-type joints, whereas the improvement of lap-type joints is limited, likely due to concentrated stress distribution and minimal geometric variation.

In the edgewise case, the peak loads of all structures decrease markedly, demonstrating that changes in the environment or loading conditions generally weaken adhesive performance. However, the double-slope joint still maintains the highest capacity (55.14 × 10^6^ N), with a smaller relative reduction than other structures, showing good stability and resistance to degradation. The next highest capacities are observed in the single-slope and double-step joints, while the single-step and lap-type joints show relatively lower performance.

It should be emphasized that the blade segment results are numerical predictions based on the modeling framework validated at the laminate level. Therefore, they are interpreted primarily as a comparative ranking under the investigated static boundary conditions rather than experimentally validated blade-level strengths.

#### 3.2.2. Consistency Analysis

**Figure 15:**
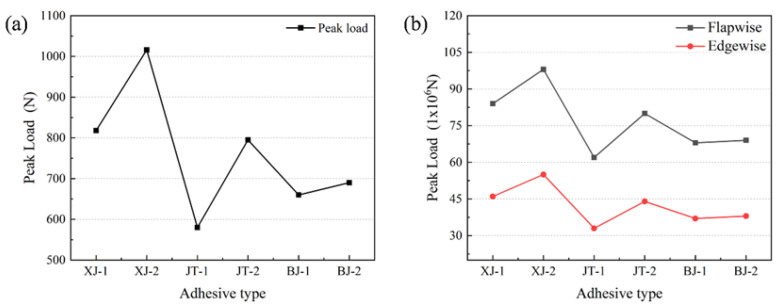
Peak loads of different adhesive structures: (**a**) line chart of CFRP laminates; (**b**) line chart of modular blades.

As shown in Figure 15, both in CFRP laminates and modular blades, the peak loads of the six adhesive structures exhibit clear structural dependence. Figure 15a presents the peak load line chart of different adhesive structures in CFRP laminates, while Figure 15b shows the peak load line chart of different adhesive structures in modular blades under flapwise and edgewise loading.

It can be observed that the single-slope joint consistently shows lower bearing capacity than the double-slope joint in all models, but significantly higher than step-type and lap-type joints. This highlights the effectiveness of slope joints in delaying interfacial debonding and improving peak load. Among step-type structures, the double-step joint generally outperforms the single-step joint, with a relatively stable load difference. Lap-type joints show relatively low peak loads, and the difference between single-lap and double-lap joints is negligible, reflecting the limitations of lap joints under three-point bending and lateral loading due to stress concentration and lower utilization of the adhesive area.

Regardless of the loading condition, Figure 15 shows that the double-slope joint consistently exhibits the highest peak load capacity. In CFRP laminates, the peak load reaches approximately 1017 N, significantly higher than other structures and showing a marked improvement over the single-slope joint. This advantage is also maintained in modular blades under both flapwise and edgewise loading, where the double-slope joint achieves the highest bearing capacity. The benefits of stress dispersion and adhesive area utilization in slope-type joints are effective under three-point bending and edgewise loading. These findings suggest that, in practical engineering applications, if modular blades are required to achieve both high load capacity and adaptability to multiple operating conditions, the double-slope joint should be prioritized. The geometric design of the joint plays a decisive role in static bearing capacity, delaying damage propagation, and enhancing adaptability to different load cases.

#### 3.2.3. Damage Evolution

**Figure 16:**
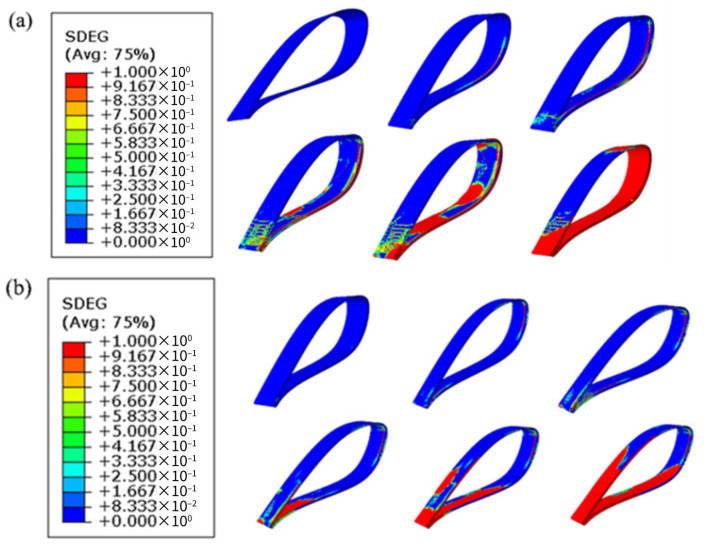
Damage evolution contours of the adhesive layer in the double-slope joint: (**a**) flapwise loading; (**b**) edgewise loading.

Compared with other configurations, the double-slope adhesive joint shows superior mechanical performance in both CFRP laminates and modular blades. To better understand the initiation and propagation of damage in the double-slope adhesive layer within blades, numerical simulations using the scalar stiffness degradation variable (SDEG) were carried out to illustrate the failure process. Figure 16a presents the damage evolution contours of the adhesive layer in the double-slope structure under flapwise loading, while Figure 16b shows the corresponding contours under edgewise loading.

In the flapwise configuration of the modular blade, damage initiation in the adhesive layer is relatively localized, but during the mid-propagation stage, the damage band rapidly spreads to both sides, eventually forming through-thickness failure across most of the adhesive layer. This process is likely dominated by uniform bending and shear, causing the interface to reach critical stress over a wide area simultaneously and leading to a steadily advancing damage band. The propagation mode is relatively uniform with a large failure area, indicating that under this condition, the interfacial stress distribution is more balanced, with faster propagation speed and wider coverage.

In the edgewise configuration, damage initiation occurs over a broader range, with multiple initiation sites appearing early at the lower surface of the lap ends and in local regions. This process may involve localized peeling or eccentric shear components, which concentrate stress in specific areas and delay global failure. However, during propagation, localized high-stress zones rapidly coalesce into an overall failure surface, presenting a concentrated and explosive failure mode. Although the final failure also penetrates the adhesive layer, the red high-damage zones are more localized, indicating that this loading condition induces stronger localized stresses.

From the two sets of SDEG contour maps, it is evident that adhesive layer damage originates from stress concentration zones at the lap ends, primarily governed by shear and accompanied by a certain degree of peeling. Ultimately, both cases exhibit through-interface failure, with failure locations strongly correlated to the mechanical loading path.

## 4. Conclusions

With the rapid growth of global wind energy demand, modular blade adhesive joints are being developed to overcome the manufacturing and transportation bottlenecks of conventional monolithic blades. This study combined numerical simulations and experimental tests to investigate the mechanical behavior, damage evolution, and failure mechanisms of CFRP laminates with six adhesive configurations under bending loads. The load-bearing capacity and damage characteristics of modular blades under flapwise and edgewise loading were also examined. The main conclusions are as follows:

(1) Load-bearing capacity of CFRP laminates

Three-point bending tests show that the double-slope joint has the best performance, with a peak load of 1017.26 N, 24.3% higher than the single-slope joint and far exceeding other structures. Proper geometric optimization can significantly improve stress distribution and load-bearing capacity.

(2) Failure mechanisms and damage evolution

The double-slope joint shows superior behavior in both interfacial failure and SDEG-based simulations. It fails mainly through cohesive damage, accompanied by matrix cracking and delamination. Step-type joints show more complex multi-path damage, while lap joints are dominated by interfacial debonding. SDEG analysis confirms that slope joints delay failure and maintain higher capacity at larger deflections by alleviating stress concentrations.

(3) Strength of modular blades

Under flapwise and edgewise static loading, for the blade segment orientation consistent with Figure 5 (right end fixed, left end loaded), the double-slope joint again performs best, with predicted peak loads of 98.24 × 10^6^ N and 55.14 × 10^6^ N, respectively, significantly higher than other joints. This ranking agrees with the CFRP laminate results, indicating the stress dispersion and debonding resistance of slope designs under the investigated static boundary conditions. The mirrored orientation (swapping fixed and loaded ends) was not simulated and may affect the peak load, which is beyond the present scope.

(4) Simulation method

The simulation method was validated by good agreement between the model and the experimental results of CFRP laminates, confirming its effectiveness. A simulation model of modular blades was then established to analyze the failure of adhesive interfaces. The model effectively reproduced the dynamic evolution process of the adhesive layer, from damage initiation to final failure, providing trend-oriented numerical guidance for blade development.

### Scope and Limitations

The above conclusions are limited to quasi-static load-bearing behavior. In practical service, wind turbine blades are primarily governed by fatigue loading and environmental actions (e.g., humidity and temperature). Moreover, the viscoelastic response of adhesives may alter damage accumulation and even the relative ranking of joint configurations; these effects require dedicated fatigue–environmental testing and modeling.

In addition, the blade-level results reported here are numerical predictions based on the validated coupon-level modeling framework, rather than experimentally validated blade segment outcomes. Uncertainties remain when extrapolating from laminate specimens to blade segments, including differences in adherend material systems, geometric details, manufacturing tolerances, bondline thickness uniformity, and boundary-condition idealizations. Mechanistically, the stress dispersion feature of the double-slope geometry may delay fatigue crack initiation; however, environmental degradation and adhesive viscoelasticity may alter crack growth rates and mixed-mode partitioning, so the ranking requires dedicated validation.

Accordingly, the joint superiority identified herein (especially that of the double-slope joint) should be regarded as valid primarily for static load-bearing capacity within the specific joint geometries and fixed parameters investigated in this study. Further optimization within the double-slope category may lead to different absolute strengths and potentially a different ranking. Its performance under fatigue loading and environmental exposure may also differ due to damage accumulation and adhesive viscoelasticity.

## Figures and Tables

**Table 1:** Mechanical properties of Araldite 2015 adhesive.

Mechanical Properties	Value
Young’s modulus E/GPa	1.85
Shear modulus G/GPa	0.56
Poisson’s ratio v	0.33
Peak normal traction $T_{n,max}$/MPa	21.63
Peak shear traction $T_{s,max}$ $T_{t,max}$/MPa	17.90
Mode I fracture energy $G_{IC}$/N/mm	0.43
Mode II fracture energy $G_{IIC}$/N/mm	4.70

**Table 2:** Selected technical parameters of the wind turbine blade.

Parameter	Value
Rated power P/MW	10
Rotor diameter D/m	205
Blade length L/m	100
Blade mass M/kg	114,172
Rated wind speed V/(m·s^−1^)	11.3

**Table 3:** Selected geometric and airfoil parameters of the blade.

Interface	Spanwise Position (%)	Chord Length (m)	Twist Angle (°)	Airfoil Type
1	0.179	7.550	13.043	Transition
2	0.196	7.622	12.912	DU99-W-405
3	0.223	7.587	12.135	DU99-W-405
4	0.248	7.487	11.350	DU99-W-350
5	0.276	7.347	10.568	DU99-W-350

**Table 4:** Material properties of glass fiber-reinforced composites and foam.

Material	E_L_/GPa	E_T_/GPa	G_LT_/GPa	_VLT_	T_FSL_/MPa	C_FSL_/GPa	τ_TU_/MPa
Unidirectional fabric	41.8	14.0	2.63	0.28	972	−702	30
Biaxial fabric	13.6	13.3	11.8	0.51	144	−213	30
Triaxial fabric	27.7	13.62	7.2	0.39	558	−457	30
Foam	0.256	0.256	0.022	0.3	5.05	−5.05	5.61

**Table 5:** Mechanical property parameters of T300 composite.

Property	Value	Unit
Longitudinal elastic modulus E_11_	125,000	MPa
Transverse/normal elastic modulus E_22_, E_33_	11,300	MPa
Longitudinal–transverse/longitudinal–normal shear modulus G_12_, G_13_	5430	MPa
Transverse–normal shear modulus G_23_	3980	MPa
Longitudinal–transverse/longitudinal–normal Poisson’s ratio ν_12_, ν_13_	0.3	-
Transverse–normal Poisson’s ratio ν_23_	0.42	-
Longitudinal tensile strength X_t_	2000	MPa
Longitudinal compressive strength X_c_	1100	MPa
Transverse tensile strength Y_t_	80	MPa
Transverse compressive strength Y_c_	280	MPa
Shear strength S	120	MPa
Normal and shear stiffness K_n,_ K_s_	100,000	N/mm^3^
Tensile failure stress σ	28.5	MPa
Shear failure stress τ	35.5	MPa
Normal critical fracture energy $G_{n}^{c}$	0.34	N/mm
Tangential critical fracture energy $G_{s}^{c}$ $,\;G_{t}^{c}$	0.38	N/mm

## Data Availability

The original contributions presented in this study are included in the article. Further inquiries can be directed to the corresponding author.

## References

[1] Martulli L.M., Diani M., Sabetta G., Bontumasi S., Colledani M., Bernasconi A. (2025). Critical review of current wind turbine blades’ design and materials and their influence on the end-of-life management of wind turbines. Eng. Struct..

[2] Peeters M., Santo G., Degroote J., Van Paepegem W. (2017). The concept of segmented wind turbine blades: A review. Energies.

[3] Kim T., Lio A.W.H.H., Lee G.H., Choi D.K., Lee S.Y. (2024). An innovative segmented blade concept with a partial pitch control for large wind turbine systems. J. Phys. Conf. Ser..

[4] Mendoza A.S.E., Yao S., Chetan M., Griffith D.T. (2022). Design and analysis of a segmented blade for a 50 MW wind turbine rotor. Wind. Eng..

[5] Verma A.S., Jiang Z., Vedvik N.P., Gao Z., Ren Z. (2019). Impact assessment of a wind turbine blade root during an offshore mating process. Eng. Struct..

[6] Baon F., Bermudo C., Trujillo F.J., Martín-Béjar S., Herrera M., Sevilla L. (2024). Adhesive bonding operations for aeronautical materials. Joining Operations for Aerospace Materials.

[7] Hart-Smith J. (2021). Aerospace industry applications of adhesive bonding. Adhesive Bonding.

[8] Guillaume J.-L. (2023). Next-generation adhesives for wind turbine blades. Adhes. Adhes. + SEALANTS.

[9] Lee D., Kim J.H., Yang S.B., Kwon D.J. (2025). Development of reprocessable structural adhesives based on covalent adaptable networks for wind turbine blade. Compos. Part B Eng..

[10] Pettersson J. (2016). Analysis and Design of an Adhesive Joint in Wind Turbine Blades. Master’s Thesis.

[11] Li Y., Liu Z., He Z., Tu L., Huang H.-Z. (2023). Fatigue reliability analysis and assessment of offshore wind turbine blade adhesive bonding under the coupling effects of multiple environmental stresses. Reliab. Eng. Syst. Saf..

[12] Venkatappagari S., Mutra R.R., Reddy D.M. (2025). State-of-the-art in adhesive joint technology: A comprehensive review of recent progress. J. Mater. Res. Technol..

[13] Zhang J., Lin G., Wang V.H. (2023). Past, present and future prospective of global carbon fibre composite developments and applications. Compos. Part B Eng..

[14] Adin H. (2017). Effect of overlap length and scarf angle on the mechanical properties of different adhesive joints subjected to tensile loads. Mater. Werkst. Und Bauteile Forsch. Prüfung Anwend..

[15] Akpinar I.A., Gultekin K., Akpinar S., Akbulut H., Ozel A. (2017). Research on strength of nanocomposite adhesively bonded composite joints. Compos. Part B Eng..

[16] Sun X., Cheng L. (2020). Prediction of failure behavior of adhesively bonded CFRP scarf joints using a cohesive zone model. Eng. Fract. Mech..

[17] Sülü I.Y. (2017). Mechanical behavior of single-lap and double-lap adhesive joined composite parts. Mater. Test..

[18] Davaasambuu K., Dong Y., Pramanik A., Basak A.K. (2025). Mechanisms and performance of composite joints through adhesive and interlocking means—A review. J. Compos. Sci..

[19] Roga S., Bardhan S., Kumar Y., Dubey S.K. (2022). Recent technology and challenges of wind energy generation: A review. Sustain. Energy Technol. Assess..

[20] Firoozi A.A., Hejazi F. (2024). Innovations in wind turbine blade engineering: Exploring materials, sustainability, and market dynamics. Sustainability.

[21] Payne C.G.T., Vuillaume A.D. (2009). Wind Turbine Rotor Blade Having Segmented Tip.

[22] Yarbrough A.A., Caruso C.D., Rodwell A.M., Kasperski D.J. (2022). A Segmented Rotor Blade for a Wind Turbine and Methods for Joining the Same. U.S. Patent Appl..

[23] Damgaard B.B., Datta V., Fukami K., Andersen P.B. (2024). Joint for Segmented Wind Turbine Blade, Segmented Wind Turbine Blade and Method for Manufacturing the Same. Patent.

[24] Johnson S.B., Chen X., Walker A.M. (2020). Segmented Wind Turbine Rotor Blade with Welded Joint. Patent.

[25] Han X., Hu M., Wang Y., Liu B., da Silva L.F.M., Guo X. (2024). Experiments and modelling of competitive failure behaviour of CFRP stepped-lap repairs with different design parameters. Thin-Walled Struct..

[26] Bidadi J., Googarchin H.S., Akhavan-Safar A., Carbas R.J.C., da Silva L.F.M. (2023). Characterization of bending strength in similar and dissimilar carbon-fiber-reinforced polymer/aluminum single-lap adhesive joints. Appl. Sci..

[27] (2014). Wind turbines—Part 23: Full-Scale Structural Testing of Rotor Blades.

[28] Mandell J.F., Samborsky D.D. (1997). DOE/MSU Composite Material Fatigue Database: Test Methods, Materials, and Analysis.

[29] Liu P.F., Liu J.W. (2022). Finite element analysis of competitive damage mechanisms of composite scarf adhesive joints by considering thickness effect. Theor. Appl. Fract. Mech..

[30] Schmandt C., Marzi S. (2025). A strain-rate-dependent cohesive zone model for peel-loaded thick and flexible adhesive layers of various geometries prone to stick–slip failure. Theor. Appl. Fract. Mech..

[31] Hu C.X. (2021). Study on the Performance of Adhesively Bonded CFRP Laminated Structures and Optimization Using a Genetic Algorithm. Master’s Thesis.

[32] (2021). Standard Test Method for Flexural Properties of Polymer Matrix Composite Materials.

